# Streptococcus agalactiae Meningitis in a Diabetic Middle-Aged Woman: A Case Report and Literature Review

**DOI:** 10.7759/cureus.110463

**Published:** 2026-06-08

**Authors:** Sean Yi Xian Tay, Chee Yik Chang

**Affiliations:** 1 Infectious Diseases, Newcastle University Medicine Malaysia, Gelang Patah, MYS; 2 Infectious Diseases, Hospital Sultanah Aminah, Johor Bahru, MYS

**Keywords:** gbs, group b streptococcus, infection, meningitis, streptococcus agalactiae

## Abstract

*Streptococcus agalactiae* (*S. agalactiae*)* *(Group B *Streptococcus*, GBS) is a well-established cause of neonatal meningitis but rarely causes meningitis in adults, accounting for only 1.3% of culture-positive cases. We report the case of a 57-year-old woman with diabetes mellitus and hypertension who presented with altered mental status, fever, and hyperosmolar hyperglycemic state. Initial investigations revealed leukocytosis, acute kidney injury, and metabolic acidosis. Cerebrospinal fluid (CSF) analysis showed lymphocytic pleocytosis, elevated protein, and low glucose. CSF culture was negative, but a multiplex polymerase chain reaction (PCR) panel detected *S. agalactiae*. The patient responded to appropriate antimicrobial therapy and supportive measures and was eventually discharged. This case highlights the potential severity of adult GBS meningitis, particularly in patients with underlying medical comorbidities. Prompt diagnosis using molecular methods is crucial for achieving favorable outcomes. A literature review is included to compare the clinical features, complications, and management strategies of adult GBS meningitis.

## Introduction

*Streptococcus agalactiae *(*S. agalactiae*)is a Gram-positive, catalase-negative coccus that typically presents in pairs or chains on a Gram stain [1]. It is also known as Group B *Streptococcus *(GBS), arising as the sole member of the Lancefield group B classification of streptococci, characterized by its unique cell-wall-associated carbohydrate, Group B antigen [2]. GBS forms small-colored colonies which cause complete hemolysis when grown on blood agar, due to the production of beta-hemolysin from the bacterium [1].

GBS is a typical organism present in the gastrointestinal and genitourinary tracts of up to 35% of asymptomatic healthy women [1]. However, GBS can become pathological when passed to newborns during delivery, resulting in severe infections such as meningitis [1]. GBS meningitis in neonates has been well-established and documented, accounting for over 86% of cases of bacterial meningitis below two months of age in the United States [3]. In comparison, GBS meningitis in adults has historically been rare, only accounting for 0.004-4.3% of adult bacterial meningitis based on cohort studies [3-6]. *Streptococcus pneumoniae*, *Neisseria meningitidis*, and *Listeria monocytogenes* have been the primary causative organisms in adult bacterial meningitis for nearly a century [7].

GBS meningitis represents a severe manifestation of invasive GBS disease, defined by the presence of GBS in sterile sites such as cerebrospinal fluid (CSF) or blood [8]. Observational studies showed that a significant proportion of patients with GBS meningitis have chronic or immunocompromising conditions, including diabetes mellitus, hepatic cirrhosis, and autoimmune disorders [3,6]. GBS meningitis can also lead to serious neurological and cardiovascular complications [9].

Diagnosis of GBS meningitis is similar to that of other bacterial meningitides, involving the correlation of clinical features with findings from blood and CSF investigations [10]. Identification of GBS is typically achieved through blood and CSF cultures, although multiplex polymerase chain reaction (PCR) panels for meningoencephalitis offer high sensitivity and specificity, particularly in culture-negative cases [10]. Benzylpenicillin remains the mainstay of treatment, with ampicillin and cephalosporins serving as effective alternatives [11].

We report a case of *S. agalactiae* meningitis in an adult woman with diabetes mellitus, complicated by severe cardiorespiratory involvement and nosocomial infections. A literature review was conducted alongside this case report, examining 34 published cases of adult GBS meningitis to compare clinical presentations, treatments, and outcomes.

## Case presentation

A 57-year-old woman with underlying hypertension and type two diabetes mellitus was referred to our emergency department from a district hospital. She had presented with three days of altered behavior, lethargy, and fever preceded by a week of reduced oral intake. She denied chest pain, palpitations, vomiting, or diarrhea, and there was no recent head trauma or seizures. Collateral history from her husband excluded recent travelling or exposure to animals. He reports them staying together in a flat, where she was previously well and functionally independent as a homemaker. She does not smoke nor consume alcohol.

Our patient was confused upon initial examination, with a fluctuating Glasgow Coma Scale (GCS) score between 11 (E3V3M5) and 13 (E4V4M5). She was oriented to place and person but not time. Vital signs were stable with a blood pressure of 133/73 mmHg, heart rate of 88 beats per minute, respiratory rate of 24 breaths per minute, oxygen saturations of 98% on 5 liters of oxygen via face mask, and a temperature of 37.6°C. General assessment revealed mild tachypnea with unremarkable respiratory, cardiovascular, and abdominal examinations. Cranial nerve assessment was limited due to poor cooperation. Neurological examination revealed normal muscle tone and reflexes in all limbs, full power (MRC: 5/5) in the upper limbs, and reduced power (MRC: 3/5) in both lower limbs. Nuchal rigidity was also noted on examination.

Initial blood investigation results are tabulated in Table 1. Our patient notably had leukocytosis with a neutrophil predominance coupled with biochemical features of severe renal impairment and a markedly elevated random blood glucose. Further testing showed a raised serum osmolality with normal serum ketone levels. An arterial blood gas analysis revealed metabolic acidosis with appropriate respiratory compensation, with significant hypoxemia and normal pCO2 levels consistent with type one respiratory failure. Our patient's clinical presentation and blood test findings are suggestive of an intracranial infection complicated by hyperosmolar hyperglycemic state (HHS) and acute kidney injury.

**Table 1:** The patient’s laboratory results

Parameters	Patient values	Reference ranges
Full blood count		
Hemoglobin	11.1 g/dL	12.2-16.2 g/dL
White blood cells	18.69 x 10^3^/μL	4.80-10.20 x 10^3^/μL
Neutrophil %	92.5%	37.0-80.0%
Lymphocyte %	4.3%	10.0-50.0%
Renal profile		
Urea	41.2 mmol/L	3.5-7.2 mmol/L
Creatinine	347 μmol/L	50-98 μmol/L
Liver function test		
Total bilirubin	13.8 μmol/L	3.4–20.5 μmol/L
Alkaline phosphatase	306 U/L	40-150 U/L
Albumin	27 g/L	35-52 g/L
Globulin	46 g/L	25-30 g/L
Alanine transaminase	16 U/L	<55 U/L
Cardiac enzymes		
Aspartate transaminase	25 U/L	5-34 U/L
Creatinine kinase	184 U/L	29-168 U/L
Lactate dehydrogenase	367 U/L	125-220 U/L
Arterial blood gas		
pH	7.36	7.35-7.45
pO_2_	48 mm Hg	80-100 mm Hg
pCO_2_	32 mm Hg	35-45 mm Hg
HCO_3_^-^	18.1 mmol/L	22-26 mmol/L
Base excess/deficit	-6.4 mmol/L	-2 to +2 mmol/L
Other tests		
Random blood sugar	39.7 mmol/L	7.8-11.1 mmol/L
Serum ketones	0.3 mmol/L	< 0.6 mmol/L
Serum osmolality	336 mOsm/kg H₂O	275-295 mOsm/kg H₂O
Urine analysis		
Glucose	3+	<50 mg/dL
Protein	3+	<10 mg/dL
Blood	2+	<0.03 mg/dL
Ketones	Negative	
Nitrites	Negative	
Leukocytes	Negative	

Initial chest radiography was clear, and a contrast-enhanced CT scan of the brain revealed no evidence of intracranial bleeding or brain abscesses. A lumbar puncture was performed on the second day of admission, with CSF analysis showing an elevated white cell count composed exclusively of lymphocytes, raised protein levels, positive globulin, and low CSF glucose. Both CSF and blood cultures showed no growth, and testing for *Cryptococcus* and *Mycobacterium tuberculosis* returned negative. CSF meningitis/encephalitis panel via Qiagen multiplex PCR subsequently detected *S. agalactiae* (Table 2).

**Table 2:** The patient’s CSF testing results CSF: cerebrospinal fluid; PCR: polymerase chain reaction

Parameters	Patient values	Reference ranges
Opening pressure	18cm H_2_0	7-18cm H_2_0
Appearance	Slightly xanthochromic, blood-stained	Clear, colorless
White blood cell count	150 (100% lymphocytes)	0-5 cells/µL
CSF glucose	2.7 mmol/L	2.7-4.4 mmol/L
CSF protein (total)	0.96 g/L	0.15-0.60 g/L
CSF globulin	Positive	
Gram stain	Numerous pus cells, no organisms	
Culture and sensitivity	No growth	
Meningitis/encephalitis panel (multiplex PCR)	Streptococcus agalactiae was detected	

Empirical antibiotic therapy included intravenous ceftriaxone 2 g twice daily and intravenous acyclovir 500 mg once daily (dose adjusted based on renal function). Following confirmation of *S. agalactiae* on CSF PCR, antibiotics were promptly switched to intravenous benzylpenicillin 2 million units every four hours with adjunctive intravenous dexamethasone 4 mg twice daily. Our patient was managed in a general medical ward, requiring intermittent hemodialysis as renal replacement therapy.

Our patient’s GCS deteriorated on her third day of admission with a score of 7 (E2V1M4). She was admitted to the intensive care unit for endotracheal intubation and mechanical ventilation. A repeat CT head scan revealed no acute abnormalities or abscesses. Following seven days of intravenous benzylpenicillin treatment, our patient developed nosocomial *Burkholderia cenocepacia* bacteremia, prompting escalation of antibiotics to intravenous meropenem. She completed a total of three weeks of effective antibiotic therapy against *S. agalactiae*.

Our patient showed marked improvement in level of consciousness and was afebrile in her second week of admission. She was weaned off mechanical ventilation successfully and was subsequently discharged after five weeks of hospital admission with plans for an outpatient follow-up.

## Discussion

*Streptococcus agalactiae*, or GBS, is a Gram-positive coccus that typically appears in pairs or chains and is characterized by complete hemolysis on blood agar [1]. It can be isolated from the genitourinary and gastrointestinal tracts of approximately one-third of healthy women [1], which correlates with its role as the leading cause of neonatal meningitis [3]. However, GBS meningitis in adults is much less common, accounting for only 1.3% of bacterial meningitis cases with positive CSF cultures [9]. Studies have shown that adult GBS meningitis often occurs in individuals with underlying comorbidities, such as diabetes mellitus or immunosuppressive conditions, and the risk increases with age [6]. Similarly, our patient’s underlying hypertension and diabetes likely predisposed her to infection. 

To better contextualize our case with current literature, we conducted a PubMed search for reported cases of adult GBS meningitis. Case series and reports of adult patients (≥18 years old) with confirmed GBS meningitis were included in our analysis. Cases involving individuals less than 18 years of age were excluded, as were those involving pregnancy or the postpartum period. We evaluated risk factors, clinical presentations, and complications while comparing treatment regimens alongside patient outcomes.

**Figure 1:**
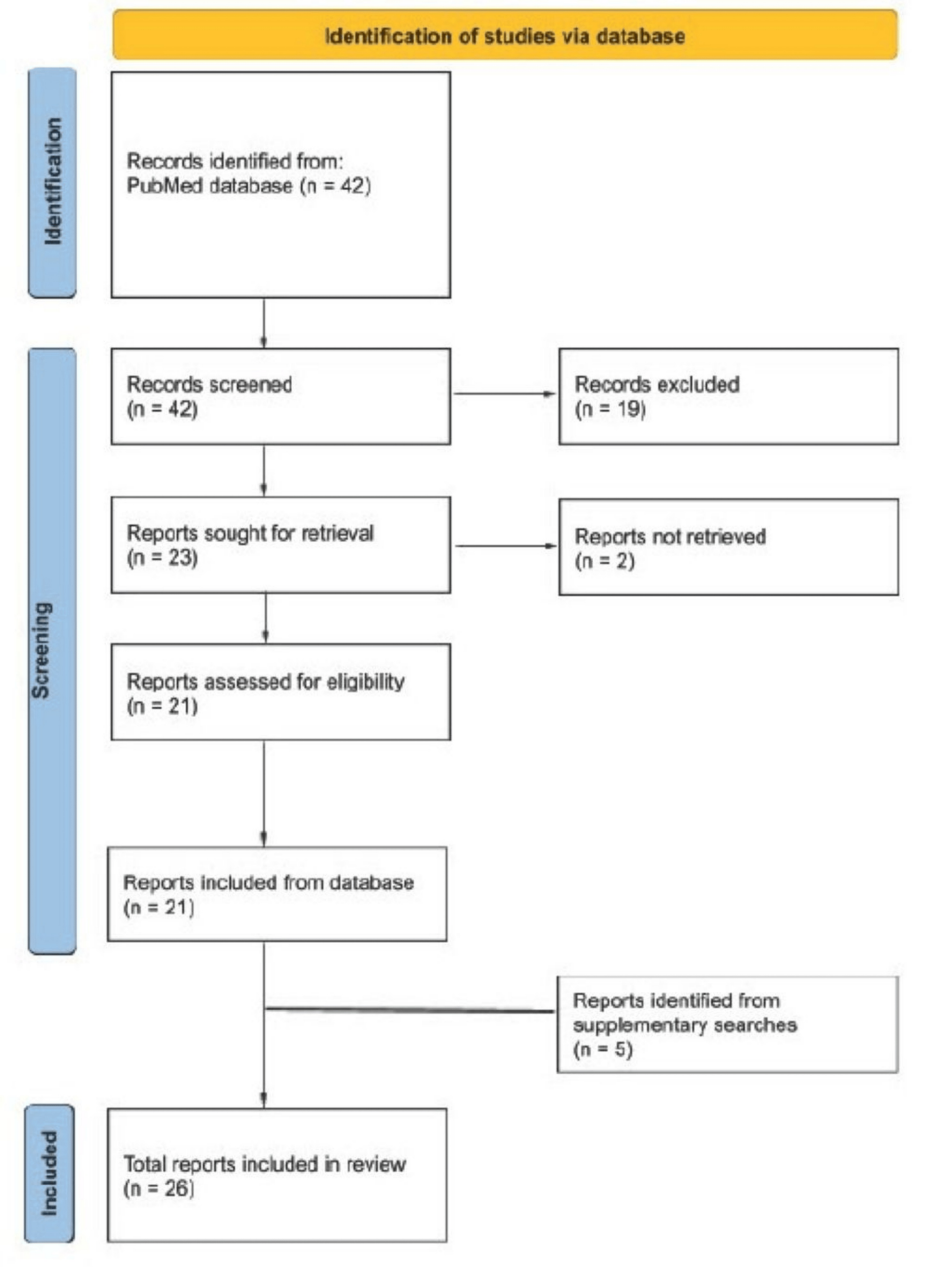
PRISMA flowchart illustrating our literature review of case reports and series PRISMA: Preferred Reporting Items for Systematic Reviews and Meta-Analyses

We performed a PubMed database search on 1 June 2025 with the following search strategy: “((GBS[Title/Abstract]) OR (group b streptococcus[Title/Abstract]) OR (streptococcus agalactiae[Title/Abstract])) AND (meningitis[Title/Abstract]) AND (adults[Title/Abstract]).” Figure 1 details our literature search with a Preferred Reporting Items for Systematic Reviews and Meta-Analyses (PRISMA) flow diagram [12]. The literature search yielded 42 articles. Of these, 19 were excluded as they did not meet our inclusion criteria, and two were excluded due to the unavailability of the full text. Additional supplementary searches identified five relevant cases, which were included. In total, 26 reports describing 34 adult cases of GBS meningitis, including our own case, were included in the review (Table 3) [13-37].

**Table 3:** Summary of published adult GBS meningitis cases (n = 34) GBS: Group B Streptococcus; CSF: cerebrospinal fluid; PCR: polymerase chain reaction; WBC: white blood cell

Author (year)	Patient demographic	Clinical presentation	CSF and blood culture findings	Treatment	Complications	Outcomes
Harburg et al. (1984) [13]	58-year-old, female; hypertension	Deafness, disorientation, and headache. Neck stiffness and truncal ataxia	Cloudy appearance with raised white cells (1400 cells/mm^3^, 90% polymorphs), raised protein, and low glucose. CSF and blood cultures grew GBS	Penicillin G for 14 days	Severe hearing impairment	Recovered with hearing complications
Dunne & Quagliarello (1993) [14]	27-year-old, male; no known medical illnesses	Fever, headache, and nuchal rigidity. Disorientation and combativeness	Cloudy appearance with raised white cells (15,000 cells/mm^3^, 96% polymorphs), raised protein, and low glucose levels. Blood cultures were negative, but CSF grew GBS	Penicillin G for 2 weeks	Deterioration of mental status prompting intubation	Recovered
	24-year-old male; recent GBS meningitis (not reported) and left mastoiditis requiring mastoidectomy	Left ear and neck pain, headache, and fever	Cloudy appearance with raised white cells (11,700 cells/mm^3^, 90% polymorphs), raised protein, and low glucose. Blood cultures were negative, but CSF grew GBS	Penicillin for 8 weeks.	None reported. CSF leak identified	Recovered
	54-year-old female; insulin-dependent diabetes mellitus and heart transplant	Nausea, vomiting, back pain, fever, and altered consciousness on the 4th day of hospitalization	Cloudy appearance, raised white cells (7300 cells/mm^3^, 100% polymorphs), raised protein levels, and reduced CSF to serum glucose ratio. Both CSF and blood cultures identified GBS	Penicillin G	Cardiorespiratory failure and brain death	Passed away
	82-year-old female; coronary artery disease, insulin-dependent diabetes, end-stage renal failure, and myelodysplasia	Chills, lethargy, and generalized seizure with fever	Cloudy, xanthochromic appearance with raised white cells (7500 cells/mm^3^, 90% polymorphs), raised protein, and reduced glucose levels. Both CSF and blood cultures grew GBS	Ampicillin and vancomycin for co-existing cellulitis and endovascular infection	Infective endocarditis	Passed away
Guerin et al. (2000) [15]	45-year-old male; HIV infection (CD4 count of 173/mm^3^)	Clinical signs of meningitis (details not reported)	Cloudy appearance with raised cell count (560 polynuclear white cells/mm^3^), raised protein, and reduced glucose levels. Both CSF and blood cultures identified GBS	Amoxicillin for 2 weeks	Coma requiring mechanical ventilation	The patient was weaned off mechanical ventilation but remained disoriented and was subsequently lost to follow-up
Agouridakis et al. (2005) [16]	17-year-old female; no known medical illnesses	Fever, headache, and vomiting. Cervical stiffness, positive Kernig’s and Brudzinski’s signs	Raised white cells (18/mm^3^, 95% neutrophils), normal protein and glucose levels. Gram stain showed gram-positive cocci. CSF and blood cultures returned GBS	Vancomycin for 15 days and penicillin	None reported	Recovered
González et al. (2013) [17]	52-year-old female; traumatic brain injury 20 years ago	Headache, neck pain, impaired consciousness, and fever	Cloudy appearance with raised cell count (8480/mm^3^, 100% polymorphs), raised protein, and low glucose levels. Gram stain was negative. CSF and blood cultures returned GBS	Ceftriaxone for 2 weeks	None reported. CSF leak identified	Recovered
Protonotariou et al. (2015) [18]	41-year-old male; systemic lupus erythematosus	Febrile diarrhea, drowsiness, headache, confusion, and cervical stiffness. Reduced GCS of 6 with mydriasis on examination	Blood cultures were negative. Post-mortem CSF analysis revealed neutrophilic WBC of 1800 cells/mm^3^, low glucose of 43 mg/dL, and elevated protein (500 mg/dL). CSF culture grew GBS	Vancomycin, ceftriaxone, and acyclovir		Passed away
Li et al. (2016) [19]	26-year-old male; no known medical illnesses	Headache, photophobia, and confusion	Cloudy appearance with raised white cell count (2140 mm^3^, 90% polymorphs), raised protein, and low glucose levels. CSF culture returned negative, but blood cultures grew GBS	Ceftriaxone for 2 weeks	None reported	Recovered
Khan (2016 [20]	72-year-old female; diabetes, hypertension, and hypothyroidism	Fever, headache, vomiting, and drowsiness. Neck rigidity and right-sided hemiparesis	Turbid appearance with leukocytes (10000 cells/µL), raised protein, and low glucose. CSF and blood cultures identified GBS	Ceftriaxone for 2 weeks	None reported	Improved
Kuzume et al. (2019) [21]	39-year-old male; no known medical illnesses	Impaired consciousness, headache, nausea, fever, and neck stiffness	Cloudy appearance with raised WBC of 1012/µL (polymorphonuclear cells 96%), raised protein (147.3 mg/dL), and glucose (44 mg/dL). CSF and blood cultures revealed GBS	Ceftriaxone and ampicillin for 2 days, and meropenem for a total of 13 days	None reported	Recovered
Germano et al. (2019) [22]	78-year-old female; diabetes, hypertension, and gout	Altered mental status and fever. Nuchal rigidity, positive Kernig’s and Brudzinski’s signs	Purulent appearance with raised white blood cells (9.107 x 10^9^/L, 98% neutrophils), raised protein, and glucose levels. CSF culture grew GBS, while blood cultures were negative	Ceftriaxone for 25 days	Resistant hypertension and epileptiform discharges	Recovered
Hirai et al. (2020) [23]	41-year-old, Male; Langerhans cell histiocytosis (on immunosuppressive therapy)	Fever and chills	Purulent appearance with elevated opening pressure, raised neutrophils (5954 cells/mm^3^), raised protein, and low glucose levels. CSF cultures grew GBS, but blood cultures were negative	Ceftriaxone, vancomycin, and dexamethasone for 4 days, followed by ampicillin for 17 days	None reported	Recovered
Al-Bayati et al. (2020) [24]	38-year-old female; gestational diabetes mellitus, and recently treated for an ear infection with amoxicillin	Altered mental status, fever, headache, and nausea	Turbid and straw-colored, glucose (59 mg/dL), raised protein (366 mg/dL), leukocytosis (17,982/µL) with raised segmented neutrophil count (83%), and low monocyte count (2%). CSF and blood cultures grew GBS	Penicillin G for 14 days	Intubated and ventilated for 5 days	Recovered
Payus et al.(2020) [25]	38-year-old female; no known medical illnesses	Fever, headache, dizziness, vomiting, and generalized tonic-clonic seizures. Neck stiffness and jolt accentuation on examination	Raised opening pressure, raised cell count (2100/µL, predominantly neutrophils), raised protein, and low glucose. CSF culture was negative, but blood cultures identified GBS	Ceftriaxone for 2 weeks	None reported	Recovered
Abdelradi et al. (2020) [26]	69-year-old female; diabetes mellitus and ovarian cancer	Progressive confusion, low-grade fever, nausea, and vomiting for five days	Leukocytosis (4500/mm^3^), raised protein levels (354 mg/dL), glucose (140 mg/dL), with a negative Gram stain. Blood cultures from admission grew GBS	Penicillin G (4 weeks) and gentamicin (2 weeks)	Endocarditis, ventriculitis, and duodenal ulcer	Recovered
Villareal et al. (2021) [27]	55-year-old, Female; Obesity, juvenile rheumatoid arthritis, and recent acute otitis media	Right-sided headache, confusion, nausea, and vomiting	Significant leukocytosis (12815/mm^3^), low glucose (39 mg/dL), and elevated protein (564 mg/dL). CSF culture showed no growth, but blood culture revealed GBS. PCR on CSF confirmed GBS	Ceftriaxone for 2 weeks	None reported	Recovered
Cahill et al. (2022) [28]	62-year-old male; diabetes, dyslipidemia, gastroesophageal reflux, and benign prostatic hyperplasia	Fever, cough, generalized weakness with intermittent headaches, and altered consciousness	Yellow-tinged appearance with WBC count of 875 x 10^6 ^cells/L (81% neutrophils), red blood cell count of 88 x 10^6^ cells/L, normal glucose, and raised protein levels. CSF cultures were negative, but blood cultures identified GBS	Ampicillin initially, later switched to ceftriaxone and metronidazole for polymicrobial infection coverage	Heart failure and eventual cardiac arrest	The patient improved after 2 weeks, but later died of heart failure
Tsalta-Mladenov et al. (2022) [29]	62-year-old female; hypertension, atrial fibrillation, and cervical cancer	Disoriented, right-sided body weakness, low back pain, and fever	Positive Pandy reaction, marked leukocytosis (2470/μl), high CSF protein (3.44g/L), and low CSF glucose (<0.6 mmol/L). GBS was isolated from both CSF and blood cultures	Triple combination of broad-spectrum antibiotics with dexamethasone	Septic cerebral embolisms	The patient remained comatose and passed away after several days
Coelho et al. (2022) [30]	66-year-old female; hypertension, dyslipidemia, overweight, and chronic alcohol consumption	Altered consciousness and fever	Pleocytosis (14 leukocytes, 50% polymorphonuclears) and elevated protein. CSF and blood cultures were positive for GBS	Ampicillin; changed to ceftriaxone after diagnosis of septic arthritis (total 6 weeks)	Septic arthritis	Recovered
Shahkarami et al. (2022) [31]	22-year-old male; no known medical illnesses	Altered consciousness and headache	Turbid appearance, leukocytosis (5500 WBCs, 10% lymphocytes, 90% neutrophils), low glucose, and raised protein. CSF culture returned positive for GBS	Ceftriaxone for 2 weeks with initial dexamethasone	Shingles	Recovered
Hudson et al. (2023) [32]	74-year-old female; metabolic syndrome, generalized anxiety disorder, and splenectomy. Recently, a COVID-19 infection (treated with monoclonal antibodies)	Cough, chest pain, and shortness of breath with normal neurological examination	Clear fluid with no nucleated cells, normal protein, and raised glucose levels. Both CSF and blood cultures were negative, but PCR on CSF revealed GBS	Ceftriaxone for a total of 2 weeks	Worsening of psychiatric symptoms, which progressed from paranoid psychosis to mania	Recovered
Ide et al. (2023) [33]	22-year-old Asian male; no underlying medical illnesses	Headache, fever, and positive signs of meningeal irritation	Pressure (315 mmH_2_O), raised cell count of 1845/µL (polymorphonuclear cells 67.9%), and glucose of 51 mg/dL. Blood and CSF cultures returned negative, but the meningitis and encephalitis panel revealed GBS	Vancomycin and ceftriaxone for 16 days with dexamethasone for the initial 5 days	None reported	Recovered
Ahmad et al. (2025) [34]	33-year-old male; Burkitt lymphoma	Recurrent vomiting, vertigo, and visual disturbances. Bilateral diplopia upon horizontal gaze	Elevated white blood cell count (420 cells/mm^3^, 80% polymorphonuclear cells), elevated protein, and reduced glucose levels. CSF culture and meningitis array identified GBS	Ampicillin for 2 weeks	None reported	Recovered
Onaka et al. (2025) [35]	74-year-old male; diabetes mellitus	Progressive gait instability and fever	Xanthochromic appearance with raised cell count (140 cells/µL), raised protein, and normal glucose levels. Blood cultures returned GBS. CSF culture was negative, but PCR and mass spectrometry identified GBS	Penicillin G for 8 weeks and gentamicin for 2 weeks due to suspected infective endocarditis	Discitis and subdural empyema	Recovered
Borriello et al. (2025) [36]	37-year-old male; benign neurinoma of the left acoustic nerve	Fever, headache, and diarrhea	Raised white blood cells (6165 cells/mm^3^, 90% neutrophils), and normal glucose levels. CSF culture and PCR identified GBS	Vancomycin and clindamycin for 10 days	None reported	Not available
	50-year-old female; obesity	Confusion	Raised white blood cells (6980 cells/mm^3^, 88.1% neutrophils), and normal glucose levels. CSF culture returned negative, but PCR identified GBS	Ceftriaxone and vancomycin for 10 days	None reported	Not available
Vidhya et al. (2025) [37]	61-year-old male; chronic alcohol use for 25 years	Fever and altered sensorium	Raised white cells (1895 cells/µL, 95% polymorphs), raised protein, and low glucose levels. CSF culture revealed GBS	Ceftriaxone and vancomycin		Passed away
	43-year-old male; hypertension	Fever, headache, altered sensorium, right hemiparesis, and slurred speech. Neck stiffness on examination	Raised protein and reduced glucose levels. CSF culture revealed GBS	Ceftriaxone and vancomycin	None reported	Recovered
	35-year-old male; history of tuberculosis and alcohol excess	Fever, headache, vomiting, neck pain, and loose stools. Neck stiffness on examination	Raised cell count (3840 cells/µL, 95% polymorphs), raised protein, and normal glucose levels. Gram stain showed Gram-positive cocci. CSF culture returned positive, and mass spectrometry confirmed GBS	Ceftriaxone and vancomycin	None reported	Recovered but lost to follow-up
	21-year-old male; chronic alcohol use	Fever, headache, vomiting, altered sensorium, and right limb weakness	Raised protein and reduced glucose levels. Gram stain showed gram-positive cocci. CSF culture returned positive, and mass spectrometry confirmed GBS	Ceftriaxone and vancomycin	None reported	Recovered
	85-year-old male; no known medical illnesses.	Fever, headache, vomiting, and altered sensorium	Raised protein and reduced glucose levels. CSF gram stain and culture returned negative. CSF PCR confirmed GBS	Ceftriaxone and vancomycin	None reported	Recovered
Our case	57-year-old female; hypertension and diabetes mellitus	Altered behavior, lethargy, and fever	Xanthochromic appearance, raised white cells (150/mm^3^, 100% lymphocytes), raised protein, and borderline low glucose levels. CSF and blood cultures were negative, but CSF PCR identified GBS	Penicillin G and meropenem (total 3 weeks)	Endotracheal intubation and mechanical ventilation	Recovered

Among the 34 reported cases of adult *S. agalactiae* meningitis, 18 patients (52.9%) were male and 16 (47.1%) were female. The majority of cases (76.5%, n = 26) occurred in individuals under 65 years of age, while eight patients (23.5%) were aged 65 years or older. Comorbidities were common, with diabetes mellitus identified in nine patients (26.5%), autoimmune or immunosuppressive conditions in six patients (17.6%), and malignancies in four patients (11.8%). Notably, eight patients (23.5%) had no known underlying medical illnesses (Table 4).

**Table 4:** Demographic and risk factors of reported Streptococcus agalactiae meningitis cases (n = 34)

Patient characteristic	Number (percentage)
Male	18 (52.9%)
Female	16 (47.1%)
<65 years	26 (76.5%)
≥65 years	8 (23.5%)
No known medical illnesses	8 (23.5%)
Diabetes mellitus	9 (26.5%)
Malignancy	4 (11.8%)
Autoimmune/immunocompromised conditions	6 (17.6%)

Clinically, GBS meningitis is indistinguishable from other forms of bacterial meningitis, often presenting with similar CSF profiles marked by neutrophilic pleocytosis [6]. Common clinical features include headache, fever, neck stiffness, and altered mental status [9], although scattered petechial lesions have also been reported in some cases [6]. This clinical presentation was consistent with our patient, who presented with fever and fluctuating levels of consciousness, with a GCS score ranging from 11 to 13. Notably, her initial CSF analysis revealed lymphocytic pleocytosis, which raised suspicion for a viral etiology. However, elevated protein and reduced glucose levels were more consistent with bacterial meningitis despite the CSF culture being negative. Ultimately, multiplex PCR confirmed *S. agalactiae* as the causative pathogen.

Diagnosis of GBS meningitis is usually established by correlating clinical presentation and test results [10]. Among the 34 cases, we noted that different investigations were used to identify GBS, namely, CSF cultures, blood cultures, and CSF PCR via meningoencephalitis panels. CSF culture was the most frequently ordered investigation (97% of cases), followed by blood culture (73.5%) and CSF PCR (26.5%). The limited use of CSF PCR (n = 9) may reflect either restricted availability or its more recent adoption into clinical practice. CSF culture identified the highest number of GBS cases (n = 24), whereas blood culture and CSF PCR identified 17 and 9 cases, respectively. 

**Figure 2:**
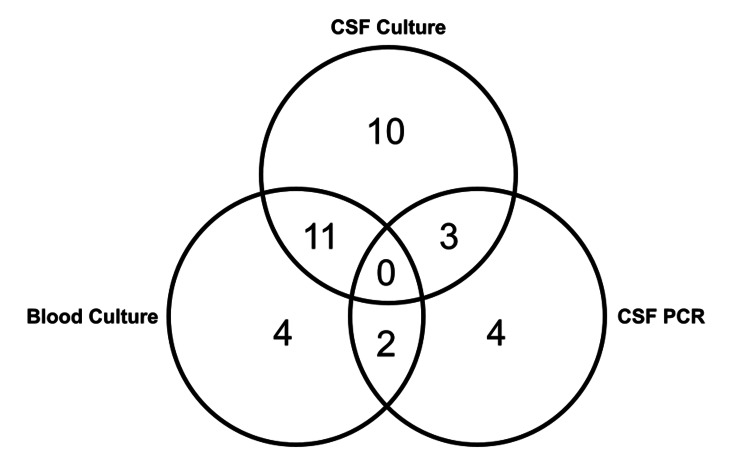
Venn diagram illustrating the overlap of positive results from CSF culture, blood culture, and CSF PCR in confirmed GBS cases (n = 34). The overlapping region between two circles represents cases where both corresponding tests identified GBS, while nonoverlapping regions represent single-modality detections. No cases tested positive across all three modalities (central intersection) CSF: cerebrospinal fluid; PCR: polymerase chain reaction; GBS: Group B *Streptococcus*

We assessed the diagnostic overlap between CSF culture, blood culture, and CSF PCR in 34 cases of GBS meningitis (Figure 2). In 18 cases (52.9%), *Streptococcus agalactiae* was detected by only one test, most often by CSF culture (n = 10), followed by blood culture (n = 4) and CSF PCR (n = 4). The remaining 16 cases (47.1%) had concordant results between two tests, but none were positive across all three. This highlights the importance of a multimodal diagnostic approach. Our study found that CSF and blood cultures often identified GBS when the other did not. CSF PCR was positive in four cases, including our own, where both cultures were negative. These findings support the complementary use of molecular and culture-based tests, especially when culture sensitivity may be reduced by prior antibiotics or poor sample quality [38].

Outcomes were reported in 32 of the 34 cases. Of these, 26 patients (76.5%) recovered, while six (17.6%) died due to the infection. All six patients with negative outcomes had significant comorbidities: previous heart transplant [14], end-stage renal failure and myelodysplasia [14], systemic lupus erythematosus [18], diabetes mellitus [14,28], cervical cancer [29], and chronic alcoholism [37]. Poor prognostic factors were noted in the majority of fatal cases, such as advanced age (≥ 65 years) [14], cardiorespiratory failure [14,28], infective endocarditis [14], and neurological complications [14,29]. While most survivors recovered without long-term complications, one case reported hearing complications as residual deficits [13]. In our case, the patient required mechanical ventilation, developed acute kidney injury necessitating intermittent dialysis, and experienced a prolonged hospital course complicated by nosocomial infections.

*S. agalactiae* has traditionally remained highly susceptible to penicillin, with penicillin G continuing to be the first-line treatment of choice [11]. Other beta-lactam antibiotics, such as ampicillin, cephalosporins, and carbapenems, are also considered effective alternatives [11]. Across our literature review, antibiotic treatment regimens varied: 12 patients received penicillin-based therapy, 16 were treated with cephalosporins, four received combination antibiotic regimens, and one case was treated with other antibiotics. The treatment approach was not reported in one case (Table 4). Most treatment regimens were for 14-21 days, whereas longer antibiotic courses were prescribed for those with infectious complications [14,26,30,35]. This is consistent with current treatment guidelines for adult GBS meningitis, which recommend penicillin G, ampicillin, amoxicillin, ceftriaxone, and cefotaxime as suitable options for 14-21 days [10,39,40].

## Conclusions

*S. agalactiae* meningitis in adults, though rare, is associated with significant morbidity and mortality. The use of sensitive molecular diagnostic modalities, such as multiplex PCR, improves prompt and accurate pathogen identification. Early administration of targeted beta-lactam antimicrobial therapy remains the cornerstone of effective treatment.

## References

[1] Hanna M, Noor A (2025). Streptococcus Group B. http://www.ncbi.nlm.nih.gov/books/NBK553143/.

[2] Lancefield RC (1934). A serological differentiation of specific types of bovine hemolytic streptococci (Group B). J Exp Med.

[3] Thigpen MC, Whitney CG, Messonnier NE (2011). Bacterial meningitis in the United States, 1998-2007. N Engl J Med.

[4] Durand ML, Calderwood SB, Weber DJ, Miller SI, Southwick FS, Caviness VS Jr, Swartz MN (1993). Acute bacterial meningitis in adults-a review of 493 episodes. N Engl J Med.

[5] Sigurdardóttir B, Björnsson OM, Jónsdóttir KE, Erlendsdóttir H, Gudmundsson S (1997). Acute bacterial meningitis in adults. A 20-year overview. Arch Intern Med.

[6] Domingo P, Barquet N, Alvarez M, Coll P, Nava J, Garau J (1997). Group B streptococcal meningitis in adults: report of twelve cases and review. Clin Infect Dis.

[7] van Ettekoven CN, Liechti FD, Brouwer MC, Bijlsma MW, van de Beek D (2024). Global case fatality of bacterial meningitis during an 80-year period: a systematic review and meta-analysis. JAMA Netw Open.

[8] Skoff TH, Farley MM, Petit S (2009). Increasing burden of invasive group B streptococcal disease in nonpregnant adults, 1990-2007. Clin Infect Dis.

[9] Kassel MN van, Bijlsma MW, Brouwer MC (20191). Community-acquired group B streptococcal meningitis in adults: 33 cases from prospective cohort studies. J Infect.

[10] (2025). WHO guidelines on meningitis diagnosis, treatment and care. https://iris.who.int/server/api/core/bitstreams/f2a6cc96-7387-42c5-9a63-f5fa4216f58c/content.

[11] Raabe VN, Shane AL (2019). Group B Streptococcus (Streptococcus agalactiae). Microbiol Spectr.

[12] Page MJ, McKenzie JE, Bossuyt PM (2021). The PRISMA 2020 statement: an updated guideline for reporting systematic reviews. BMJ.

[13] Harburg TD, Leonard HA, Kimbrough RC 3rd, Jones SR (1984). Group B streptococcal meningitis appearing as acute deafness in an adult. Arch Neurol.

[14] Dunne DW, Quagliarello V (1993). Group B streptococcal meningitis in adults. Medicine (Baltimore).

[15] Guerin JM, Mofredj A, Leibinger F, Ekherian JM, Raskine L (2000). Group B streptococcus meningitis in an HIV-positive adult: case report and review. Scand J Infect Dis.

[16] Agouridakis P, Ioannidou E, Dalezios M, Panagopoulou V, Drandakis P (2005). "Honey moon" meningitis. Emerg Med J.

[17] González B, Labatut T, Soto A, Fica A, Castro M (2013). Acute bacterial meningitis by Streptococcus agalactiae in a non pregnant woman associated to a cerebrospinal fluid leak: a case report (Article in Spanish). Rev Chilena Infectol.

[18] Protonotariou E, Arampatzi A, Ourailoglou V, Diza E, Skoura L (2015). An unusual case of Streptococcus agalactiae meningitis in a patient with sys-temic lupus erythematosus. Hippokratia.

[19] Li LQ, Cheema S, Goel N (2016). Group B streptococcal meningitis in a previously healthy man. BMJ Case Rep.

[20] Khan FY (2016). Streptococcus agalactiae meningitis in adult patient: a case report and literature review. Case Rep Infect Dis.

[21] Kuzume D, Morimoto Y, Kinboshi M, Yoshida T, Yamasaki M (2019). A rare case of Streptococcus agalactiae meningitis in previously healthy adult (Article in Japanese). Rinsho Shinkeigaku.

[22] Germano N, Sibbel MG, Summerfield D, Pitzenberger A (2019). Group B streptococcus meningitis complicated by periodic lateralising epileptiform discharges in an elderly patient with type 2 diabetes mellitus. BMJ Case Rep.

[23] Hirai J, Kinjo T, Haranaga S, Fujita J (2020). A case report of cerebral meningitis caused by penicillin-non-susceptible group B Streptococcus in an immunocompromised adult patient. Infect Drug Resist.

[24] Al-Bayati A, Douedi S, Alsaoudi G (2020). Meningitis from invasive Streptococcus agalactiae in a healthy young adult. IDCases.

[25] Payus AO, Clarence C, Azman Ali R (2020). Group B streptococcal meningitis in a healthy young woman: a case report. Int J Gen Med.

[26] Abdelradi A, Murphy A, Ahasic AM (2020). Invasive Streptococcus agalactiae causing meningitis, ventriculitis, and endocarditis in a non-pregnant adult. Cureus.

[27] Villareal K, Goslin A, Bajracharya H (2021). Group B Streptococcus meningitis associated with acute otitis media in an adult patient. Am J Case Rep.

[28] Cahill JA, Li C, Wong PH (2022). Group B streptococcal leptomeningitis, ventriculitis, right cerebellitis, and cerebritis in an immunocompetent patient. J Assoc Med Microbiol Infect Dis Can.

[29] Tsalta-Mladenov ME, Dimitrova VM, Georgieva DK, Andonova SP (2022). Streptococcus agalactiae meningitis presented with cerebral infarction in adult patient-clinical case and review. Neurol India.

[30] Coelho T, Pacheco M, Mendes T, Valente J, Gil P (2022). Invasive Streptococcus agalactiae disease with meningitis and septic arthritis in a non-pregnant patient. Cureus.

[31] Shahkarami F, Fallah Tafti M, Alizadeh M, Foroughi A, Bayati R (2022). An unusual case of Group B streptococcal meningitis with concomitant varicella-zoster virus infection in a previously healthy male. Cureus.

[32] Hudson A, Bobo D, Rueda Prada L, Dumic I, Petcu E, Cardozo M, Shweta F (2023). Mania: an atypical presentation of probable Streptococcus agalactiae meningoencephalitis. IDCases.

[33] Ide R, Kubota T, Ohtomo A, Ohtomo M, Watanabe G, Tsukita K, Suzuki Y (2024). Streptococcus agalactiae meningitis in an immunocompetent adult: a case report and literature review. Intern Med.

[34] Ahmad I, Abbas S, Anjum A, Nizamuddin S, Parveen A (2025). Streptococcus agalactiae meningitis in an adult: a case report. Cureus.

[35] Onaka J, Fukushima T, Yoshida A, Leedy N, Kobayashi T, Tomoto K, Aoki K (2025). Atypical progression of Group B Streptococcus infection: subdural empyema in an adult with diabetes mellitus. IDCases.

[36] Borriello G, Fusco G, Greco F (2025). First report of Streptococcus agalactiae meningitis in a non-pregnant adult in Italy. Microorganisms.

[37] Vidhya T, Akshay K, Veenakumari HB, Nagarathna S, Samaddar A (2025). Alert and aware: rising cases of community-acquired group B Streptococcus meningitis in immunocompetent adults. Indian J Med Microbiol.

[38] Phillips RJ, Watanabe KM, Stowell JR, Akhter M (2019). Concordance between blood and cerebrospinal fluid cultures in meningitis. Am J Emerg Med.

[39] Chang CY (2023). Pneumococcal meningitis and myocarditis in a splenectomized patient. J Glob Infect Dis.

[40] (2025). Meningitis (bacterial) and meningococcal disease: recognition, diagnosis and management. https://www.nice.org.uk/guidance/ng240/chapter/Recommendations.

